# Prevalence of Livestock-Associated MRSA ST398 in a Swine Slaughterhouse in Guangzhou, China

**DOI:** 10.3389/fmicb.2022.914764

**Published:** 2022-06-23

**Authors:** Xiaoshen Li, Longfei Xie, Honghao Huang, Zhi Li, Guihua Li, Peng Liu, Danyu Xiao, Xucai Zhang, Wenguang Xiong, Zhenling Zeng

**Affiliations:** ^1^Guangdong Provincial Key Laboratory of Veterinary Pharmaceutics Development and Safety Evaluation, College of Veterinary Medicine, South China Agricultural University, Guangzhou, China; ^2^National Risk Assessment Laboratory for Antimicrobial Resistance of Animal Original Bacteria, South China Agricultural University, Guangzhou, China; ^3^Guangdong Laboratory for Lingnan Modern Agriculture, South China Agricultural University, Guangzhou, China; ^4^College of Veterinary Medicine, China Agricultural University, Beijing, China

**Keywords:** swine slaughterhouse, multi-drug resistance, MRSA ST398, *spa* typing, whole-genome sequencing, public health

## Abstract

Livestock-associated methicillin-resistant *Staphylococcus aureus* (LA-MRSA) is an important zoonotic microorganism that is increasingly causing public health concern worldwide. The objective of this study was to determine the transmission and occurrence of MRSA in a slaughterhouse environment and evaluate its antimicrobial resistance and genetic characterization. In this study, we conducted a comprehensive epidemiological survey of *S. aureus* by *spa* typing and whole-genome sequencing (WGS) of samples obtained from the pork production chain, the environment, and community residents. To clarify the evolutionary relationships of MRSA sequence type (ST) 398 in this study and global isolates, 197 published whole-genome sequences data of MRSA ST398 strains were downloaded from the GenBank database and included in the phylogenetic analysis. A total of 585 porcine samples (snout and carcass swabs), 78 human nasal samples, and 136 environmental samples were collected. The MRSA isolates were detected at higher frequencies in samples from swine (15.0%) than carcasses (10.0%), slaughterhouse workers (8.0%), community residents (0%), and environment samples (5.9%). The *spa* typing results showed that t571 accounted for a higher proportion than other *spa* types. Closely related isolates from the samples of swine, slaughterhouse workers, carcasses, carrier vehicle, and surrounding fishpond water indicate that MRSA ST398 strains may spread among swine, humans, and the environment. MRSA ST398-t571 isolates were genetically different from global strains, except for two Korean isolates, which showed genetic closeness with it. In addition, a MRSA ST398 isolate recovered from an infected patient in Europe differed by only 31 SNPs from the airborne dust-associated strain isolated in this study, thereby suggesting potential transmission among different countries. Antimicrobial susceptibility testing results demonstrated that 99.0% (96/97) of MRSA and 95.1% (231/243) of methicillin-sensitive *S. aureus* (MSSA) showed multidrug-resistant (MDR) phenotypes. According to WGS analysis, the *poxtA*-carrying segment (IS*431mec*-*optrA*-IS*1216-fexB*-IS*431mec*) was reported in MRSA ST398 isolates for the first time. The coexistence of *cfr* and *optrA* in a plasmid was first detected in MRSA ST398. The potential transmission of MRSA among humans, animals, and the environment is a cause for concern. The emergence and transmission of LA-MRSA ST398 with high levels of resistance profiles highlight the urgent need for LA-MRSA surveillance.

## Introduction

Methicillin-resistant *Staphylococcus aureus* (MRSA) is an important pathogen that causes community- and hospital-acquired infections (Larsen et al., 2015; Nakaminami et al., 2020). Postoperative secondary infections caused by multidrug-resistant (MDR) MRSA make clinical treatment difficult (Van Duin and Paterson, 2016; Bassetti et al., 2020). Swine is an important host for the MRSA strain. Previous studies highlighted the potential zoonotic risks associated with MRSA carriage in swine farmers. Such carriage may be a consequence of frequent contact with infected animals, causing public health concern worldwide (Armand-Lefevre et al., 2005; Kinross et al., 2017).

In 2005, the Netherlands reported a case of livestock-associated MRSA (LA-MRSA) infection in a 6-month-old infant residing on a swine farm with sequence type (ST) 398 (Voss et al., 2005). According to the data from the GenBank database, we found that MRSA ST398 has increasingly spread worldwide and is widespread in Europe. It is currently found in more than 20 countries, including Germany, Denmark, Switzerland, Belgium, Netherlands, France, Italy, United Kingdom, Greece, Spain, Czech Republic, Poland, United States, and Canada. The sources are varied, but the predominant ones are swine and humans. In particular, the application of whole-genome sequencing (WGS) has revealed potential drivers for LA-MRSA ST398 dissemination, such as trading of colonized swine, contaminated transport vehicles, and colonized human (Grøntvedt et al., 2016; da Silva et al., 2017; Sieber et al., 2018). Notably, *S. aureus* protein A (*spa*) t571 is more strongly connected with human infections than animal colonization strains (Bonnet et al., 2018; Wardyn et al., 2018). Methicillin-sensitive *S. aureus* (MSSA) ST398-t571 was detected in nine families from the Dominican Republic living in New York; these families had no contact with livestock (Bhat et al., 2009). Furthermore, it was disseminated among detainees and patients in America (Davies et al., 2011; David et al., 2013). ST398-t571 MSSA isolates caused fatal necrotizing pneumonia and bloodstream infections in the United States and Europe (Rasigade et al., 2010; Bonnet et al., 2018; Wardyn et al., 2018).

The prevalence of MDR phenomenon in MRSA strains also attracted global attention. Oxazolidinones have never been approved for veterinary use in any country worldwide, but linezolid-resistant MRSA isolate has been reported in animal hosts. Linezolid is a last resort antimicrobial agent used to treat serious infections in humans caused by MDR Gram-positive bacteria, such as vancomycin-resistant enterococci, MRSA, and penicillin-resistant pneumococci (Bozdogan and Appelbaum, 2004). To date, three acquired genes, namely, *cfr* (Li S.-M. et al., 2018; Sun et al., 2018), *optrA* (Sun et al., 2018), and *poxtA* (Chen et al., 2021) conferring linezolid resistance have been mainly described in staphylococci of livestock origin. Aside from mediating linezolid resistance, these genes could confer reduced susceptibility or resistance to other antimicrobial agents (Shen et al., 2013; Wang Y. et al., 2015; Antonelli et al., 2018). Considering that they are frequently located in plasmids or other mobile genetic elements, they could be transferred between species and genera (Shen et al., 2013; Wang J. et al., 2015; Sun et al., 2018; Chen et al., 2021).

Unlike the widespread use of ST398 type of LA-MRSA strains in European and North American countries, LA-MRSA-ST9 predominates in China (Chuang and Huang, 2015; Wang J. et al., 2015; Li S.-M. et al., 2018). Previous studies showed that ST398 type of MSSA strains was frequently colonized in slaughter pigs in northeast China (Yan et al., 2014), while LA-MRSA ST398 strains were mainly isolated from humans in China (Song et al., 2017; Chen et al., 2020). In the last several years, MRSA ST398 has been identified sporadically in swine farms (Li W. et al., 2018; Zou et al., 2021), pork (Li et al., 2021), milk (Cui et al., 2020), and monkey feces (Tang et al., 2021) in China. Continued monitoring for MRSA in food-producing animals is urgently required. Comprehensive studies on the transmission of LA-MRSA among swine, humans, and the environment in swine slaughterhouses in China are few. To gain insight into the prevalence and transmission of LA-MRSA and MSSA in a typical swine slaughterhouse, *spa* typing, WGS, and bioinformatic analysis were conducted. Samples were collected from swine, carcass, environment, slaughterhouse workers, and neighboring community residents in Guangzhou, China.

## Materials and Methods

### Sampling Information

A slaughterhouse with a capacity of 3,000 swine in Guangzhou, Guangdong, China, was selected for sampling. A total of 799 samples from swine, carcasses, and humans, as well as samples from their surrounding environmental samples were collected from November 2020 to April 2021. The environment samples included airborne dusk (800–130 cm off the ground; *n* = 60), effluent (∼30 ml; *n* = 5), sludge (∼30 g; *n* = 5), workstations (surface swabs; *n* = 5), vehicles carrying swine (surface swabs; *n* = 5), river water (∼30 ml; *n* = 5), fishpond water (∼30 ml; *n* = 5), farmland soil, and vegetables (∼30 g; *n* = 46). Air samples were collected using a stationary method by placing the plates with an open lid inside the slaughterhouse area. Swine snout (*n* = 535), human nasal (slaughterhouse workers, *n* = 50; community residents, *n* = 28), and carcass swabs (*n* = 50) were collected (Supplementary Table 1). The swine samples were mainly from Guangdong, Hunan, Guangxi, and Jiangxi Provinces. Samples from individual pigs were processed separately. The slaughterhouse workers and neighboring residents signed an informed consent form and were asked to agree to nose swabbing. Information details of the volunteers are summarized in Supplementary Table 2.

### Isolation and Identification of MSSA and MRSA

The samples were soaked in 7.5% sodium chloride broth (Land Bridge, Beijing, China) and cultured at 37°C for 12 h. Then, a loopful of the broth was streaked onto chromogenic *S. aureu*s agar (Huankai Microbial, Guangdong, China). One colony with typical *S. aureus* morphology was selected from each sample and streaked onto Mannitol salt agar (Huankai Microbial, Guangdong, China) to obtain a purified isolate colony. Each isolate colony was identified by polymerase chain reaction (PCR) using the *nuc* gene to detect *S. aureus* and the *nuc* and *mecA* (or *mecC*) genes to detect the MRSA isolate (Zhang et al., 2004; Harrison et al., 2013).

### Antimicrobial Susceptibility Testing

Antimicrobial susceptibility was tested using the agar dilution method according to the guidelines of Clinical and Laboratory Standards Institute guidelines (CLSI, 2018). Tigecycline were assessed by broth microdilution as recommended by the European Committee on Antimicrobial Susceptibility Testing (EUCAST Version 6.0) (EUCAST, 2016). *S. aureus* ATCC 29213 was set as the quality control strain. The antimicrobials were amoxicillin (AMO), oxacillin (OXA), penicillin (PEN), ceftiofur (CEF), cefoxitin (FOX), gentamicin (GEN), amikacin (AMI), doxycycline (DOX), tigecycline (TIG), florfenicol (FFC), erythromycin (ERY), tilmicosin (TMI), rifampicin (RIF), vancomycin (VAN), clindamycin (CLI), tiamulin (TIA), ciprofloxacin (CIP), enrofloxacin (ENR), fosfomycin (FOS), linezolid (LZD), tedizolid (TZD), and sulfamethoxazole-trimethoprim (SXT).

### Molecular Typing and Whole-Genome Sequencing

All identified MSSA and MRSA isolates were subjected to *spa* typing by PCR amplification and sequencing of the polymorphic region of the *spa* gene (Harmsen et al., 2003). *Spa* type was assigned using *spa*-plugin in BioNumerics v7.6 (Applied Maths, Sint-Martens-Latem, Belgium). Minimum spanning trees based on *spa* types were constructed in BioNumerics v7.6 (Applied Maths, Sint-Martens-Latem, Belgium). Based on *spa* type, source, and antimicrobial resistance (AMR), 50 MRSA isolates were selected for WGS (Supplementary Tables 4, 6). The bacterial DNA was extracted using the HiPure Bacterial DNA Kit (Magen, Guangzhou, China). Illumina NOVAseq 6000 sequencing platform (Novogene Company, Beijing, China) and PacBio RS II sequencing platform (Biochip Company, Tianjin, China) were used for WGS. SMRTbell library (10–20 kb in size) was prepared by the ligation of hairpin adaptors at both ends, and the resulting library was selected according to size using Blue Pippin with a 7–10 kb cutoff for Pacbio sequencing. Illumina Novaseq sequences were assembled using CLC Genomics Workbench 10 (CLC Bio, Aarhus, Denmark). Pacbio sequences were assembled using a hierarchical genome assembly process (Chin et al., 2013). The assembled Pacbio sequences were corrected by using Burrows-Wheeler Aligner’s Smith-Waterman Alignment software to ensure that their integrity was in accordance with those of Novaseq sequences (Li and Durbin, 2010). The genome assemblies of MRSA ST398 isolates were deposited in the GenBank and were registered with BioProject number PRJNA793117. The plasmid pSA159 carrying *cfr* and *optrA* in GD4SA159 and *poxtA*-positive strain GD4SA108 were annotated by RAST annotation server^1^ (Overbeek et al., 2014). Multi-locus sequence type (MLST), acquired resistance genes, and Staphylococcal cassette chromosome *mec* (SCC*mec*) were identified against MLST (Aanensen and Spratt, 2005), ResFinder (Zankari et al., 2012), and SCC*mec*Finder (Kaya et al., 2018) databases (accessed on 16 June 2020) using a mapping approach implemented in SRST2 (Inouye et al., 2014).

To clarify the genetic relatedness of MRSA ST398 in this study and global isolates, 197 published whole-genome sequences data of MRSA ST398 strains were downloaded from the GenBank database and included for analysis (Supplementary Table 3). Based on the draft genome sequences, a core-genome single nucleotide polymorphism (SNP)-based maximum-likelihood (ML) phylogenetic tree was constructed. The genome of MRSA ST398 isolate (GenBank accession number: JAASKV000000000) was used as a reference. The tree was constructed using Parsnp in the Harvest package (Treangen et al., 2014) with default parameter settings and was annotated on the EvolView website https://evolgenius.info. A comparison of the genetic context was generated using Easyfig 2.1 (Sullivan et al., 2011) and BLAST Ring Image Generator (BRIG) (Alikhan et al., 2011).

### Statistical Analysis

Descriptive and comparative analyses were performed in Graphpad Prism 8.0 (Graphpad Software, La Jolla, CA, United States), and statistical analyses were performed in SPSS 20.0 (IBM Corp., Armonk, NY, United States). Fisher’s exact test was used to test whether differences in drug resistance phenotypes among human-, environment-, and swine-associated isolates were significant, and also evaluated between MRSA and MSSA isolates.

## Results

### Prevalence and Antimicrobial Susceptibility of MRSA and MSSA

**FIGURE 1:**
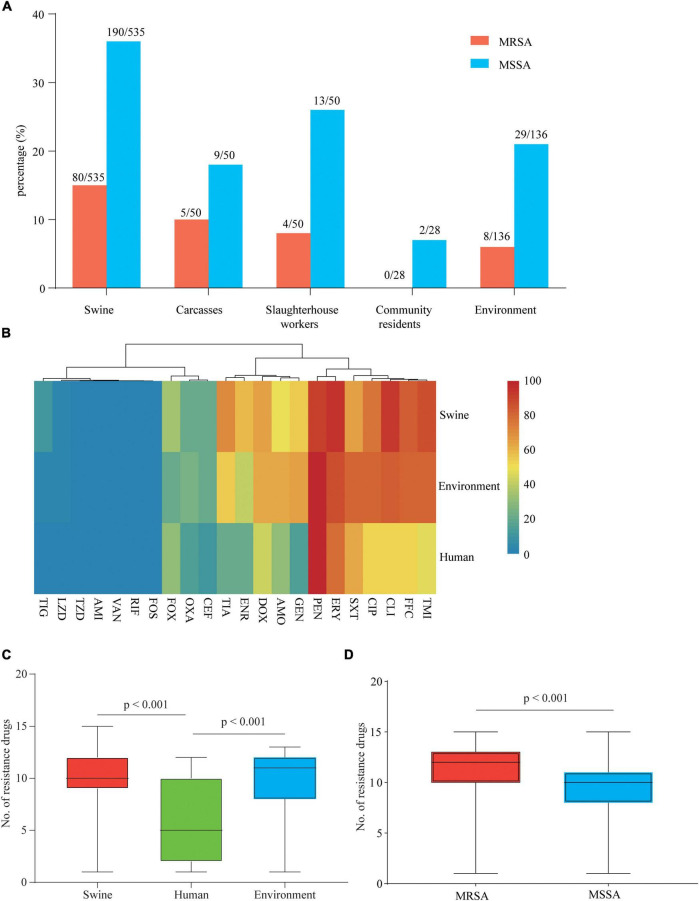
Prevalence and drug resistance of isolates recovered from samples from various origins. **(A)** Prevalence of methicillin-susceptible *Staphylococcus aureus* (MSSA) and methicillin-resistant *S. aureus* (MRSA) in samples from various origins along and around a slaughterhouse in Guangzhou, China. **(B)** Each cell in the heat map indicates the percentage of strains with resistance to a particular drug. **(C)** The bar graph indicates the total number of drugs with resistance within each swine, human, and environment-associated isolate. **(D)** The bar graph indicates the total number of drugs with resistance within each MSSA and MRSA isolate. The numbers of swine, human, and environment-associated strains were 284, 19, and 37, respectively. The numbers of MSSA and MRSA isolates were 243 and 97, respectively.

Ninety-seven MRSA and 243 MSSA isolates were collected from 799 samples (Supplementary Table 1). MRSA isolates were detected in swine (15.0%, 80/535), carcass (10.0%, 5/50), slaughterhouse workers (8.0%, 4/50), and environmental samples (5.9%, 8/136), whereas no MRSA-positive isolate was found in the samples from community (Figure 1A). MSSA-positive isolates were detected in swine (35.5%, 190/535), carcasses (18.0%, 9/50), slaughterhouse workers (26.0%, 13/50), community residents (7.1%, 2/28), and environmental samples (21.3%, 29/136) (Figure 1A).

Antimicrobial susceptibility tests of 340 isolates were performed using 22 antimicrobials (Supplementary Table 4). According to the results, 99.0% (96/97) of MRSA and 95.1% (231/243) of MSSA showed MDR phenotypes. Swine- and environment-associated isolates were resistant to a larger number of antimicrobial agents compared with human-associated isolates (median 10, 11 vs. 5; *p* < 0.001) (Figure 1C). The swine- and environment-associated isolates showed high resistance levels to florfenicol, erythromycin, tilmicosin, and clindamycin (> 80%). The trimethoprim/sulfonamide resistance rate of environment-associated isolates was significantly higher than that of isolates from swine or human samples (*p* < 0.05). All isolates from different sources showed high resistance to penicillin (> 90%) but low resistance to tigecycline, linezolid, fosfomycin, and rifampicin (< 11.47%). All isolates were susceptible to amikacin, vancomycin, and Acetazolamide (Figure 1B and Supplementary Table 5). Compared with MSSA isolates, MRSA isolates were resistant to most of the antimicrobial agents (median 12 vs. 10; *p* < 0.001) (Figure 1D). The majority of isolates recovered in the study showed a MDR phenotype.

### *Spa* Typing of MRSA and MSSA

**FIGURE 2:**
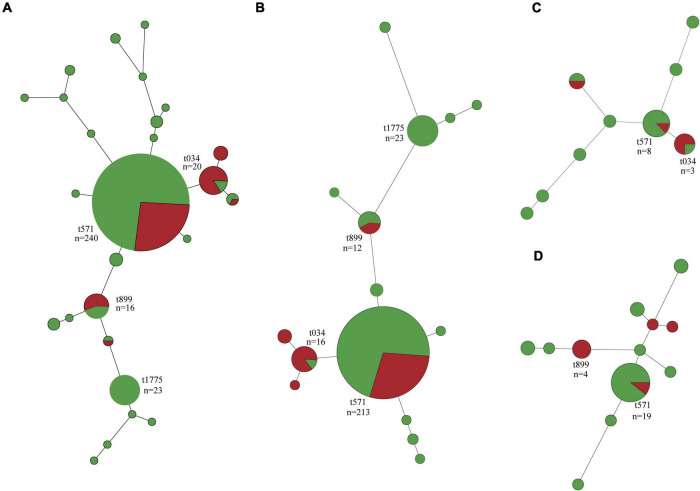
Minimum spanning tree of methicillin-susceptible *Staphylococcus aureus* (MSSA, *n* = 243) and methicillin-resistant *S. aureus* (MRSA, *n* = 97) isolates by *spa* type. **(A)** All isolates. **(B)** Isolates of swine origin. **(C)** Isolates of human origin. **(D)** Isolates of environmental origin. Each node represents a single *spa* type. The size of the node is proportional to the number of isolates represented by a said node. Branch lengths between nodes are proportional to the number of alleles that differ between the two linked nodes. Selected nodes are labeled with corresponding *spa* type and number of isolates represented. Red nodes, MRSA; green nodes, MSSA.

In total, 26 *spa* types were identified among the 340 *S. aureus* isolates, with MSSA isolates (25 *spa* types) showing greater diversity than MRSA (six *spa* types) and three isolates were not typed (Figure 2A and Supplementary Table 4). Besides t571 (64.9%, 63/97), t034, t899, t114, and t1793 were also detected in MRSA isolates. In addition to t571 (72.8%, 177/243), t034, t899, t114, t1793, and t011 were also detected in MSSA isolates (Figure 2A).

Swine-associated MRSA isolates were dominated by t571 (71.4%, 60/84); *spa* type t034 and t899 accounted for a small proportion (Figure 2B). The human- and environment-associated MRSA isolates primarily had t034 (50%, 2/4) and t899 (50%, 4/8), respectively (Figures 2C,D). MSSA t571 clonal lineage predominated in swine, human, and environment-associated isolates (Figure 2).

### Whole-Genome Phylogenetic Analysis of MRSA ST398

Among 50 MRSA isolates sequenced, ST398 was the dominant clonal lineage (84.0%, 42/50). Most MRSA isolates belonged to ST398-t571 (42.0%, 21/50), followed by ST398-t034 (30%, 15/50), ST398-t011 (10%, 5/50), ST9-t899 (14.0%, 7/50), and one ST1-t114 isolate associated with human.

**FIGURE 3:**
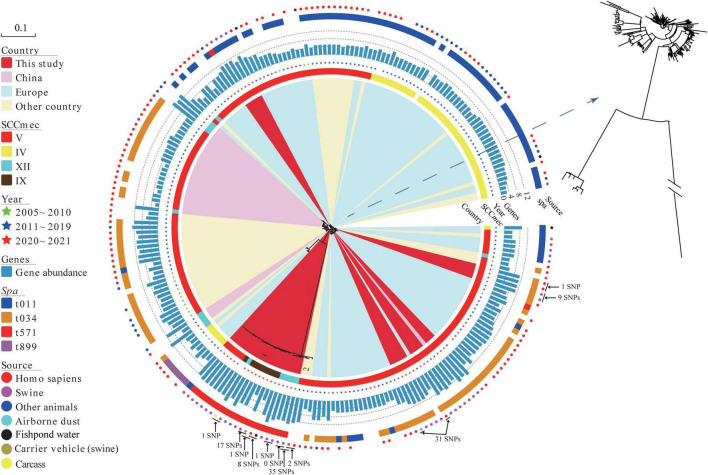
The maximum likelihood (ML) phylogenetic tree of 42 methicillin-resistant *Staphylococcus aureus* (MRSA) ST398 isolates from China based on single nucleotide polymorphisms in the core genomes. A total of 197 global MRSA ST398 isolates were included for comparison. Regions were indicated by leaf color. Sources and years of the isolates were indicated by circles and stars in different colors, respectively. SCC*mec* and *spa* types were indicated by strips in different colors. The branch of Block 2 represents Korean isolates. The total number of antibiotic resistance genes in each isolate was represented by the length of a blue bar chart. Details of the genes of each isolate are given in Supplementary Table 7. The core of phylogenetic tree was amplified for a clearer display.

Phylogenetic analyses revealed that MRSA ST398 was mainly clustered into three clades, corresponding to clonal lineage identified as SCC*mec* V-t034, SCC*mec* V-t011, and SCC*mec* IV-t011 (Figure 3). Notably, SCC*mec* were diversified in MRSA ST398-t571 clonal lineage, such as SCC*mec* V, IX, and XII subtypes, which were predicted with a template coverage of less than 60%. These isolates differed in 6,893 core genome SNPs. Interestingly, the isolates (block 1) found in this study were genetically different from global strains, except for the Korean strains (block 2) that showed close genetic relationship with the abovementioned isolates. Swine-associated Korean isolate PJFA-521M (GenBank accession number: SRKD00000000) was closely related to human-associated Korean isolate PJFH-522M (GenBank accession number: RKRI00000000) (2 SNPs difference) and swine-associated Chinese isolate (HN2SA18) (35 SNPs difference) (Figure 3). Furthermore, a strain OP17 (GenBank accession number: VKBM00000000) isolated from an infected patient in Europe differed by only 31 SNPs from the airborne dust-associated isolate (GDK3SA10) isolated in this study. The potential transmission of MRSA among swine, human, carcass, carrier vehicle, and fishpond water was observed around the slaughterhouse. Swine-associated isolate GDA3SA01 was closely related to GD4SA98 recovered from carrier vehicle (swine) (17 SNPs difference). Meanwhile, GD4SA98 was closely related to the GD4SA24 recovered from a slaughterhouse worker (1 SNP difference). GD4SA24 was closely related to GD4SA93 recovered from the surrounding fishpond water (with a difference of 8 SNPs). GD4SA24 was closely related to swine-associated isolate GDA3SA01 (with a difference of 16 SNPs). Swine-associated isolate GD4SA116 was closely related to GD4SA159 recovered from carcass (with a difference of 1 SNP). This was also observed among slaughterhouse workers, as GD3SAM4 was closely related to GD3SAT1 (with a difference of 1 SNP).

### Distribution of Antibiotic Resistance Genes in MRSA ST398

Antibiotic resistance genes (ARGs) in MRSA ST398 isolates (42 sequenced isolates in this study and 197 records retrieved in the GenBank database) are displayed in Supplementary Figure 1 (Supplementary Table 7). A total of thirty-three ARGs grouped into nine types were found in samples from swine, human, and environment. Figure 3 shows that the abundance of resistance genes of sequenced MRSA ST398 isolates in this study was higher than that of global isolates. Notably, the prevalence rates of penicillin/methicillin resistance gene *blaZ* and tetracycline resistance gene *tetM* in MRSA ST398 were 95.82% and 88.28%, respectively. In addition, the detection rates of tetracycline resistance gene *tet*(K), trimethoprim resistance gene *dfrG*, lincomycin resistance gene *lnu*(B), macrolides-lincosamides-streptogramin B (MLS_B_) resistance gene *erm*(C), lincosamides-pleuromutilins-streptogramins A resistance gene *lsa*(E), aminoglycoside resistance genes *aac(6′)-aph(2″)*, *ant(9)-Ia*, and *aadD*, and florfenicol resistance gene *fexA* ranged from 20% to 50%. The multiresistance genes *cfr*, *optrA*, and *poxtA* were detected in MRSA ST398. The coexistence of the *cfr* and *optrA* genes was detected in a plasmid of GD4SA159 with oxazolidinone sensitivity that was isolated from carcass. In addition, the swine-associated isolate GD4SA108 carried *poxtA* along with the resistance genes *aac(6′)*-*aph(2″)*, *aadD*, *ant(6)*-*Ia*, *blaZ*, *dfrG*, *erm*(C), *erm*(T), *fexA*, *fexB*, *lnu*(B), *lsa*(E), *mecA*, *tet*(L), *tet*(M), and *qacG*. Alarmingly, two slaughterhouse worker-associated isolates (GD3SAM4 and GD3SAT1) carried the *cfr* gene, as well as the *ant*(9)*-Ia*, *blaZ*, *dfrG*, *erm*(A), *erm*(C), *fexA*, *mecA*, *tet*(K), *tet*(M), and *vga*(E). The two isolates showed an MDR phenotype, which indicated resistance to beta-lactams, tetracyclines, florfenicol, erythromycin, tilmicosin, clindamycin, tiamulin, and ciprofloxacin.

### The Coexistence of *cfr* and *optrA* in a Plasmid

**FIGURE 4:**
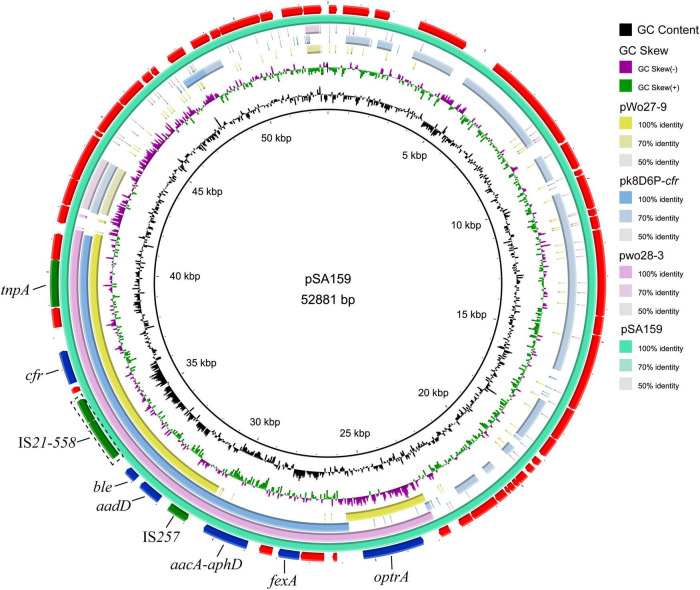
Ring comparison of plasmid pSA159 (GD4SA159) using BRIG. Arrows indicate the positions and directions of gene transcription. Blue and green arrows indicate resistance genes and mobile genetic elements (MGEs), respectively. Genes encoding hypothetical proteins and proteins for plasmid stability are depicted in red.

To characterize the genetic environment of the *cfr* and *optrA* genes, plasmid pSA159 (GenBank accession no. CP090408) from MRSA ST398 isolate GD4SA159 was completely sequenced. It was 52,881 bp in length and had an average GC content of 38.0%. A total of 53 open reading frames (ORFs) coding for proteins of > 50 amino acids were identified. Except for the 20 ORFs encoding hypothetical proteins with no defined function, the products of the remaining 36 ORFs exhibited identities ranging from 33.0% to 100.0% to proteins with known functions, such as AMR, plasmid replication, and conjugative transfer or transposition. The plasmid pSA159 with the *optrA-fexA-aacA-aphD-aadD-ble-cfr* resistance gene cluster was subjected to a comparative analysis with other plasmids (Figure 4). The 16,956 bp segment between *optrA* and *tnpA* showed 99.68% identity with the *cfr*- and *optrA*-carrying region of plasmid pwo28-3 from pig-associated *S. sciuri* (KT601170). The segment truncated *optrA* displayed 99.8% identity with the corresponding region of plasmid pk8D6P-*cfr* from duck-associated *S. sciuri* (CP065793). The segment between *tnpA* and IS*257* showed 99.96% identity with the corresponding region of plasmid pWo27-9 from *S. sciuri* (KX982169).

### The Genetic Context of *poxtA*

**FIGURE 5:**
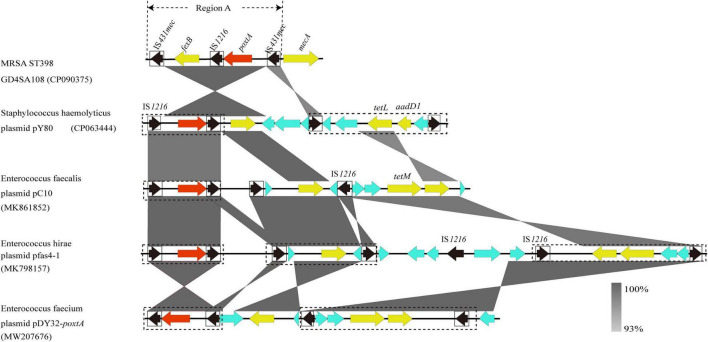
Schematic presentation of the genetic context of *poxtA*-flanking regions in the chromosomal DNAs of the isolate GD4SA108 compared with the corresponding regions of other strains in the NCBI database. Regions of >93% homologies are shaded gray. Differently colored arrows indicate the direction of transcription of the different genes. The *poxtA* gene is shown as a red arrow, and yellow arrows indicate other resistance genes. Genes with unknown or other functions are depicted in light blue. MGEs are indicated as black dotted boxes. Insertion sequences are indicated as black boxes, with the black arrow inside the box showing the transposase gene. Region A could form a circular intermediate.

The genome of MRSA-ST398 GD4SA108 (GenBank accession no. CP090375) containing the *poxtA* gene was completely sequenced and was 2,864,441 bp in length. It had an average GC content of 32.0%. As shown in Figure 5, the genetic context of *poxtA* from plasmids pY80, pC10, pfas4-1, and pDY32-*poxtA* was commonly flanked by two copies of IS*1216* elements in the same orientation. By contrast, the *poxtA* gene from chromosomal genome of GD4SA108 was flanked by two copies of IS*431mec* elements in the same orientation. It could form a circular intermediate (7,270 bp, region A) that contained IS*1216* and *fexB* genes (Figure 5). Plasmids pC10, pfas4-1, and pDY32-*poxtA* from *Enterococcus faecalis*, *E. hirae*, and *E. faecium* carried the *poxtA* gene and tetracycline resistance genes *tetM* and *tetL*. Plasmid pY80 from *S. haemolyticus* contained *tetL* and *aadD1* aside from the *poxtA* gene. The results suggested that *poxtA* gene may be transmitted with other drug resistance genes, especially tetracycline-resistant genes.

## Discussion

MRSA ST9 is a common clonal lineage among swine-associated MRSA in China (Zhou et al., 2018). In this study, fewer MRSA ST9 isolates were found in swine. In contrast, the prevalence of MRSA ST398, which is typically associated with swine and farmers in Europe, was detected in the slaughterhouse. Phylogenetic analyses revealed that MRSA ST398 was predominantly associated with the t571 circulating in the slaughterhouse. This finding differed from those of previous studies, in which t011 and t034 were commonly associated with MRSA ST398 globally (Hansen et al., 2019; Pirolo et al., 2019; Schnitt et al., 2020). The swine contaminated with MRSA ST398 was probably raised in a farm setting before being transported to the slaughterhouse, and this assumption was supported by the occurrence of MRSA ST398-t034/t571 in a pig farm in southern China (Li W. et al., 2018). Additionally, the potential spread was predicted among swine, workers, carcass, carrier vehicle, and surrounding fishpond water, as supported by the analysis of SNP difference (Bartels et al., 2015; Schürch et al., 2018; Bouchami et al., 2020). A previous study on the drivers of LA-MRSA CC398 demonstrated that swine movements were crucial for the spread of MRSA CC398 (Sieber et al., 2018). As a means of transporting for swine, vehicles that test positive for MRSA ST398 were highly likely to contaminate the transport route, leading to widespread infection. In addition, the carcasses transported to the pork market were positive for MRSA ST398, thereby indicating a potential food safety risk. Slaughterhouse workers had a higher detection rate of MRSA ST398 compared with community residents, perhaps due to the prolonged exposure to slaughterhouse conditions. Although it was not detected in the samples from residents, MRSA ST398 was detected in the nearby fishpond and airborne dust, suggesting that residents exposed to the environment were still at risk of infection. The suggestion was corroborated by a previous report (Bos et al., 2016). The infected slaughterhouse workers, carrier vehicles, fishponds, and carcasses could serve as a source for the transmission of MRSA ST398 in the community. However, large-scale sampling and further studies should be conducted to confirm this.

Phylogenetic analysis of MRSA ST398 suggested that infected human or swine may drive the spread of MRSA ST398 among different countries. The diversity of colonizing sources suggested their potential role as reservoirs for transmission. Half of the sequenced MRSA ST398 isolates showed difference in evolutionary relationship (block 1, *spa* type t571), indicating the development of diversity in the population of MRSA ST398 isolates. MSSA ST398-t571 was relatively rare among livestock isolates (Lienen et al., 2020) and was commonly associated with human infection. Amazingly, *spa* type t571 was the dominant clonal lineage in MRSA and MSSA isolates in the slaughterhouse (Bonnet et al., 2018; Wardyn et al., 2018). The virulence levels of MRSA- and MSSA-t571 isolates in this study were unclear. The frequent reports of invasive infections caused by *S. aureus* ST398-t571 isolates suggested that persistent genotypic surveillance of *S. aureus* strains in humans and animals should be practiced (Song et al., 2017; Chen et al., 2020). The prevalence of MRSA ST398 in the slaughterhouse may be closely related to foreign pig trading and traveling. The details of pig trading and history of human travel abroad could not be identified exactly. Thus, further studies are needed to clarify the reason for the prevalence of MRSA ST398 lineage.

The MRSA carriage among swine, carcass, slaughterhouse workers, and environmental samples in this study was higher than that in samples from slaughterhouses from Shandong (fatteners, 8.2%, 7/85; carcasses, 1.0%, 1/91; slaughterhouse workers, 0%, 0/16; environmental samples, 1.9%, 1/52) (Sun et al., 2019). The difference in MRSA carriage could be due to differences in the location and size of the slaughterhouse. The high prevalence of MRSA carriage in this slaughterhouse might result from the beta-lactam antibiotic usage pressure in animal production (Qiao et al., 2018). Antibiotic resistance is a serious threat to global public health. The resistance phenotypes of swine-associated isolates were generally more severe than those in human-associated isolates (e.g., resistance to florfenicol, gentamicin, doxycycline, clindamycin, tilmicosin, tiamulin, enrofloxacin, and ciprofloxacin) (Figures 1B,C). The differences in AMR phenotype between swine- and human-associated isolates could be due to differences in the type and amount of antimicrobial used. The analyses of AMR genotypes of MRSA ST398 showed that the extremely high detection of *tetM* (88.28%) was consistent with the characteristic of tetracycline resistance of CC398 clonal lineage (Ward et al., 2014), suggesting that heavy tetracycline use in the swine industry may have promoted the dissemination of this clonal lineage (Moodley et al., 2011).

Oxazolidinones are essential drugs used to address the MDR of Gram-positive bacteria. The linezolid resistance genes *cfr*, *optrA*, and *poxtA* are of concern, because they are often located in mobile genetic elements (MGEs), which could transmit among bacteria by horizontal gene transfer. The coexistence of *cfr* and *optrA* in a plasmid was first detected in MRSA ST398, although it has been reported in *S. sciuri* and *E. gallinarum* (Li et al., 2016; Fan et al., 2017; Coccitto et al., 2022). The *poxtA* gene is commonly flanked by IS*1216* element (Antonelli et al., 2018; Chen et al., 2021). However, the novel genetic context of *poxtA* gene flanked by two copies of IS*431mec* elements was identified, thereby increasing the awareness of the spread of *poxtA* gene. These *cfr*, *optrA*, and *poxtA*-carrying plasmids or fragments may be disseminated among MRSA in the slaughterhouse and possibly spread to MRSA of human origin. The *cfr*, *optrA*, and *poxtA* genes lead to resistance to chloramphenicol and florfenicol aside from linezolid (Long et al., 2006; Wang Y. et al., 2015; Antonelli et al., 2018). As the use of chloramphenicol in food animals was banned in China in 2002, florfenicol appears to be the only antimicrobial agent to select *cfr*, *optrA*, and *poxtA* from isolates of food animal origin. Thus, rational use of florfenicol in food-producing animals is urgently needed. In addition, SCC*mec* elements play a crucial role in the spread of methicillin resistance and evolution of MRSA. SCC*mec* V and SCC*mec* IV types among CC398-MRSA were prevalent worldwide (Wulf et al., 2008; Kadlec et al., 2009; Lienen et al., 2020). Compared with global isolates, SCC*mec* IV lineage isolate was absent, and the SCC*mec* V lineage dominated in MRSA ST398 isolates sequenced in this study. Interestingly, the types of SCC*mec* XII and IX were predicted in MRSA ST398-t571 isolates. These results indicated a rapid evolution of SCC*mec* elements in MRSA ST398-t571, which was similar to the findings from a previous study (Ji et al., 2020).

## Conclusion

This study provided a longitudinal investigation of the presence of MRSA in a swine slaughterhouse in China. MRSA ST398 may spread among swine, humans, and the environment in the slaughterhouse, and it showed high levels of resistance profiles. This is the first report of the coexistence of *cfr* and *optrA* genes in a plasmid in MRSA ST398 isolates. The *poxtA*-carrying segment (IS*431mec*-*optrA*-IS*1216-fexB*-IS*431mec*) was reported in MRSA ST398 isolates for the first time. The *spa* typing results confirmed that t571 clonal linage dominated in MRSA or MSSA strains. This study underscores the importance of surveillance for MRSA ST398 in swine and indicates a high likelihood for the spread of MRSA ST398 from the swine production chain to human.

## Data Availability Statement

The datasets presented in this study can be found in online repositories. The names of the repository/repositories and accession number(s) can be found in the article/Supplementary Material.

## Ethics Statement

The study was approved by the South China Agriculture University (SCAU) Animal Ethics Committee. All animals were sampled under authorization from the Institutional Animal Care and Use Committees (IACUCs) of SCAU. Written informed consent was obtained from the individual(s) for the publication of any potentially identifiable images or data included in this article.

## Author Contributions

XL wrote the first draft of the manuscript. XL, WX, and ZZ contributed to the conception and design of the study. LX, HH, ZL, GL, PL, DX, and XZ performed the sampling and statistical analysis. All authors contributed to manuscript revision, read, and approved the submitted version.

## Conflict of Interest

The authors declare that the research was conducted in the absence of any commercial or financial relationships that could be construed as a potential conflict of interest.

## Publisher’s Note

All claims expressed in this article are solely those of the authors and do not necessarily represent those of their affiliated organizations, or those of the publisher, the editors and the reviewers. Any product that may be evaluated in this article, or claim that may be made by its manufacturer, is not guaranteed or endorsed by the publisher.

## Footnotes

1
https://rast.nmpdr.org/
