# Supplementary figures and images for: Prevalence of Livestock-Associated MRSA ST398 in a Swine Slaughterhouse in Guangzhou, China
Source: Front Microbiol. 2022 Jun 23;13:914764. doi: 10.3389/fmicb.2022.914764 (PMC9260045; doi:10.3389/fmicb.2022.914764)

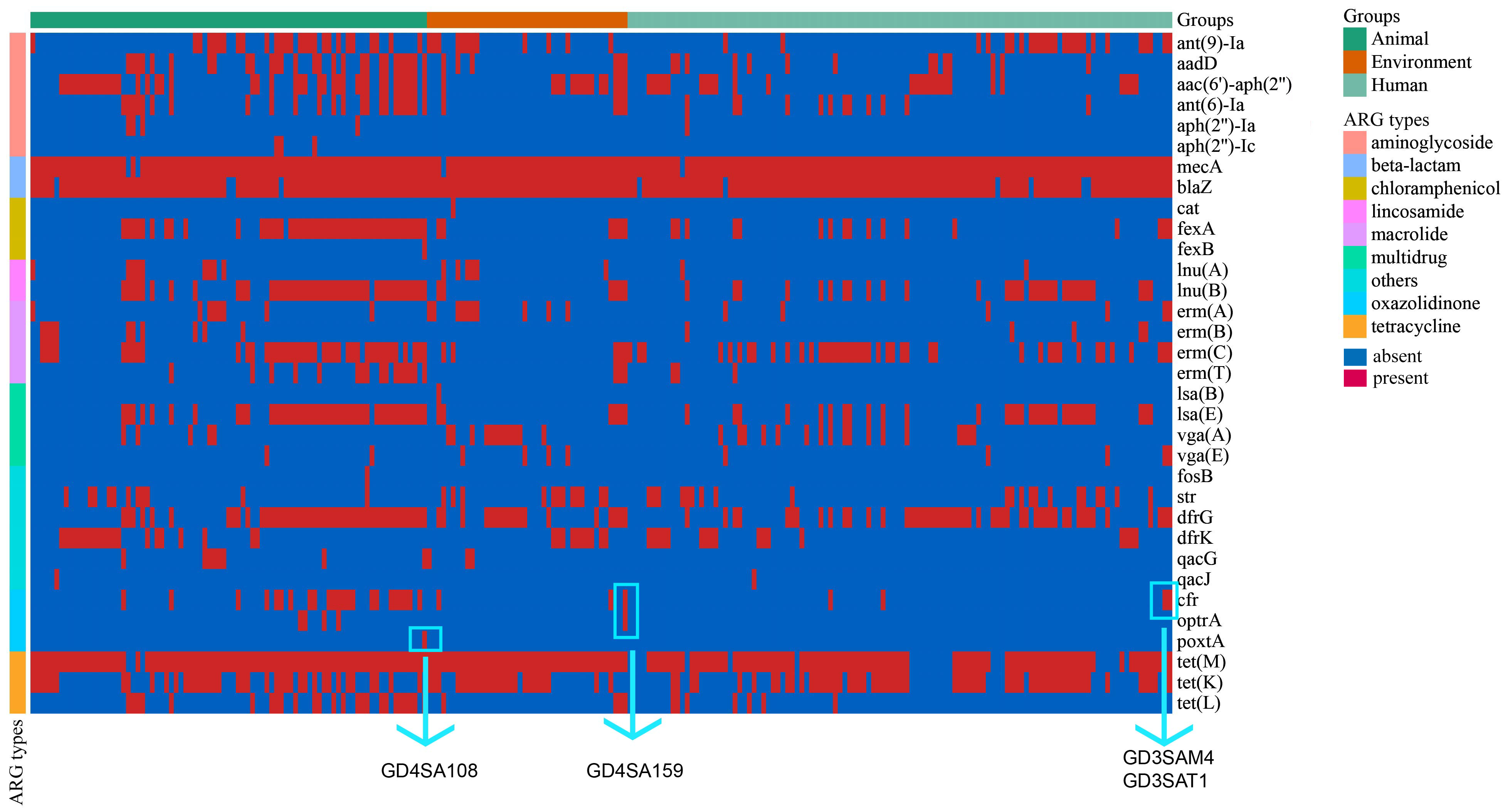

Supplement: Supplementary file 2 [file Image_1.TIF]
